# Natural Language Processing of Radiology Reports to Assess Survival in Patients with Advanced Melanoma

**DOI:** 10.3390/cancers17091595

**Published:** 2025-05-07

**Authors:** Jeeban P. Das, Jordan Eichholz, Varadan Sevilimedu, Natalie Gangai, Danny N. Khalil, Michael A. Postow, Richard K. G. Do

**Affiliations:** 1 Department of Radiology, Memorial Sloan Kettering Cancer Center, New York, NY 10065, USAdok@mskcc.org (R.K.G.D.); 2 Department of Epidemiology and Biostatistics, Memorial Sloan Kettering Cancer Center, New York, NY 10017, USA; 3 Department of Medicine, Memorial Sloan Kettering Cancer Center, New York, NY 10065, USA; khalild@mskcc.org (D.N.K.); postowm@mskcc.org (M.A.P.); 4 Department of Medicine, Weill Cornell Medical College, New York, NY 10065, USA

**Keywords:** melanoma, immunotherapy, natural language processing, NLP, oncology

## Abstract

**Background/Objectives**: To use natural language processing (NLP) to extract large-scale data from the CT radiology reports of patients with advanced melanoma treated with immunotherapy and to determine whether liver metastases affect survival. **Methods**: Patient criteria (M1 disease subclassified into M1a, M1b, or M1c) as well as alternative criteria (M1 with advanced melanoma, imaged with CT chest, abdomen, and pelvis from July 2014–March 2019) were included retrospectively. NLP was used to identify metastases from CT reports, and then patients were classified according to American Joint Committee on Cancer (AJCC) staging disease subclassified into M1L+ or M1L−, indicating whether liver metastases were present or not). Statistical analysis included constructing Kaplan–Meier survival curves and calculating hazard ratios (HRs). **Results**: 2239 patients were included (mean age, 63 years). Whether using AJCC or alternative criteria, overall survival (OS) was poorest for M1L+ (entire cohort median OS, 0.69 years [95% CI: 0.60–0.82]; immunotherapy cohort median OS, 1.4 years [95% CI: 0.92–2.0]) compared to M1L− (entire cohort median OS, 1.8 years [95% CI: 1.4–2.2]; immunotherapy cohort median OS; M1L−, 2.9 years [95% CI: 2.3–3.9]). The median HR for M1L+ (median HR, 5.35 [95% CI: 4.59–6.24]) was higher than that for M0 (*p* < 0.001). The median HR for M1L+ (median HR, 2.13 [95% CI: 1.65–2.64]) was higher than that for M0 (*p* < 0.01). **Conclusions**: Patients with advanced melanoma, particularly those with liver metastases, demonstrated inferior survival, even when treated with immunotherapy.

## Simple Summary

Advanced melanoma therapeutic outcomes have improved markedly with immunotherapy, but with variable response across patients with different metastatic patterns. Understanding the impact of specific sites of metastatic disease on survival in advanced melanoma, in particular understanding whether liver metastases have a deleterious effect, has great clinical significance. Natural language processing (NLP) allows for text extraction from a large imaging dataset to evaluate the impact of the pattern of metastatic spread. We identified 2239 patients with advanced melanoma and CT imaging using NLP and classified them according to AJCC staging criteria as well as alternative criteria indicating whether liver metastases were present (M1L+) or not (M1L−). Whether using AJCC or alternative criteria, overall survival (OS) was poorest for the M1L+ group (median OS 0.69 years and 1.4 years for the entire cohort and immunotherapy-treated subset, respectively) versus 1.8 years and 2.9 years for the M1L− group. NLP can rapidly evaluate the prognosis of melanoma patients with different metastatic patterns, confirming inferior OS seen in patients with hepatic metastases.

## 1. Introduction

Since the advent of immune checkpoint blockade (ICB) with ipilimumab (anti-CTLA-4) and later pembrolizumab and nivolumab (anti-PD-1), the treatment of advanced melanoma has improved markedly, achieving a median overall survival (OS) of up to 39 months [1,2,3,4,5]. In addition, targeted therapies directed at *BRAF*, a gene mutated in 50% of melanomas and responsible for activating the MAPK/ERK-signaling pathway implicated in melanomagenesis [6], have demonstrated highly encouraging results in phase I, II, and III trials [3,4,7,8]. Even molecular testing to determine *BRAF* mutation status is now a standard of care for patients with advanced melanoma [9,10]. Notwithstanding these advances, liver metastases resulting from advanced melanoma remain difficult to treat, due in part to the induction of T-cell–mediated systemic immune tolerance, as shown in preclinical studies [11]. In addition, clinical studies have shown that liver metastases may result in inferior OS [12,13]. It remains unclear, however, whether liver immune tolerance mechanisms contribute to worse OS outcomes in patients with advanced melanomas receiving immunotherapy.

The extraction of specific pertinent text from large imaging data archives to evaluate the impact of the pattern of metastatic spread on survival outcomes using natural language processing (NLP) has grown in popularity [14,15,16,17,18,19,20,21]. For example, accurate NLP models have been developed for the large-scale labeling of CT reports, identifying patterns of metastatic spread and their impact on survival outcomes with high accuracy [14,15]. However, to date, the application of NLP to extrapolate survival data from radiology reports in patients with advanced melanoma has been limited.

The aim of this study was to use NLP to extract large-scale data from the CT radiology reports of patients with advanced melanoma in order to determine whether the site of metastases affects survival, particularly whether liver metastases have a detrimental effect on survival.

## 2. Materials and Methods

### 2.1. Ethical Approval

This retrospective study was approved by the institutional review board at Memorial Sloan Kettering Cancer Center. Consecutive CT examinations of the chest, abdomen, and pelvis (CT CAP) performed in patients with melanoma between 1 July 2014, and 1 July 2019, were obtained from the institutional database. The text from structured reports included findings related to the lungs/airways, pleura, thoracic nodes, liver, spleen, adrenal glands, abdominopelvic nodes, pelvic organs, peritoneum, and bones [14].

### 2.2. Classification of the Extent of Metastatic Disease

The NLP model used for data analysis was developed using Python v3.6, as described previously [14]. To determine whether the site of metastases in patients with advanced melanoma has an impact on OS, the NLP model was used to generate metastases labels based on CT CAP reports, first identifying patients with and without metastatic disease (M1 and M0, respectively), starting from the date of the first CT scan with M1 disease.

Then, patients with M1 disease were classified according to American Joint Committee on Cancer (AJCC) staging criteria, which subclassifies M1 disease as follows: M1a (distant metastatic disease to the skin, soft tissue including muscles, and/or non-regional nodes), M1b (distant metastatic disease to the lung with or without M1a sites of disease), and M1c (distant metastatic disease to non-central nervous system (CNS) visceral sites with or without M1a or M1b sites of disease). Patients were not subclassified into those with M1d disease (distant metastatic disease to the CNS with or without M1a, M1b, or M1c sites of disease), as the NLP model was limited to analyzing CT body imaging reports. Since nodal metastases could not be distinguished by the NLP model as involving regional vs. distant nodes, all nodal metastases identified by the NLP model were presumed to involve the distant nodes (M1a). Since many patients had more than one CT, metastatic disease to a particular organ was considered present if it was identified on the baseline study.

In addition, as an alternative to the AJCC staging criteria, patients with M1 disease were subclassified based on whether they have M1 disease spread to the liver (M1L+) or not (M1L−). To determine whether there are differences in the OS of patients with and without liver metastases resulting from advanced melanoma, the subset of patients who were treated with immunotherapy at any time during their clinical course was identified, and these patients were then classified based on AJCC staging criteria as well as the alternative criteria subclassifying M1 disease into M1L+ or M1L−. In this subset, analysis was also performed, specifically stratifying those patients whose *BRAF* mutation status was available. *BRAF* mutation status was assessed using Memorial Sloan Kettering-Integrated Mutation Profiling of Actionable Cancer Targets (MSK-IMPACT), a hybridization assay for targeting all exons and selected introns of over 300 key cancer genes, including *BRAF*, in formalin-fixed, paraffin-embedded tumors, as detailed in prior reports [22,23,24].

### 2.3. Statistical Analysis

Kaplan–Meier curves were constructed using R 3.6.3 (R Core Team 2017) for patients stratified by AJCC M1 substages (M0, M1a, M1b, M1c) and by the presence of liver metastases (M1L+, M1L−). The log-rank test was used to assess the differences in OS between the M subcategories. Cox regression models were used to calculate hazard ratios with time-to-death as the dependent variable and M status as the stratifying variable using the mentioned classifications. Kaplan–Meier survival curves were used to visualize the differences in survival of the classifications. The type I error rate was set to 0.05 (α). 10-year cumulative incidence rates for each metastatic organ were also estimated, with 95% confidence intervals calculated using the Greenwood formula [20].

## 3. Results

### 3.1. Patient Characteristics

A total of 2239 patients with advanced melanoma were identified using NLP. The mean patient age at the time of the first CT CAP scan was 63 years. The median follow-up time was 1.8 years, and the censoring rate was 52% (1172 out of 2239).

### 3.2. Survival Outcomes of Advanced Melanoma Classified According to AJCC Staging Criteria

**Figure 1:**
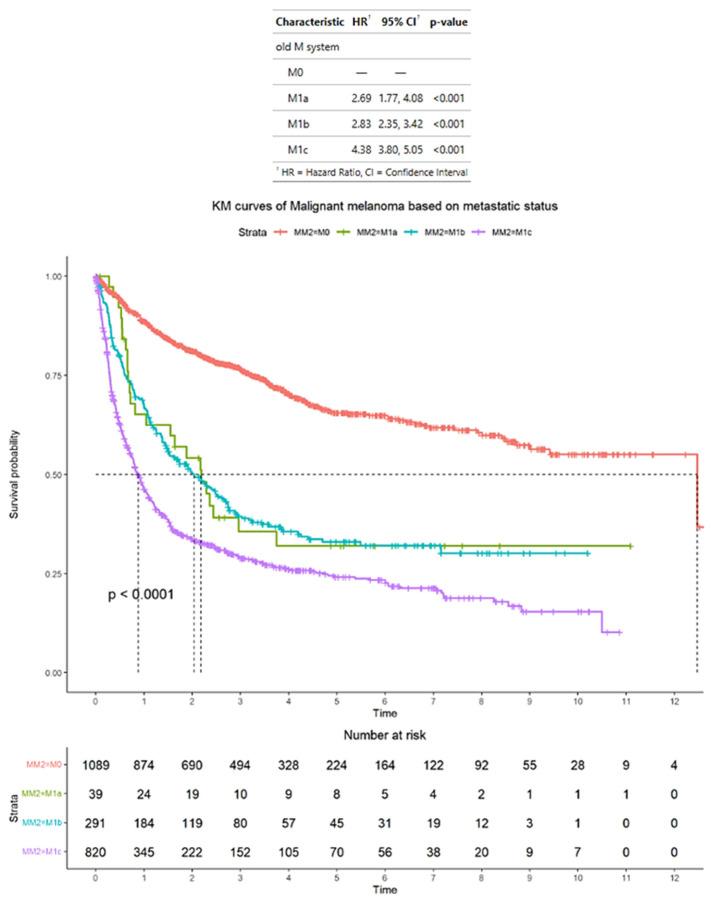
Survival outcomes in the entire sample of patients with advanced melanoma who were classified according to American Joint Committee on Cancer staging criteria. Time is measured in years.

According to the AJCC staging criteria, of the total of 2239 patients with advanced melanoma, 1089 patients had M0 disease, 39 patients had M1a disease, 291 patients had M1b disease, and 820 patients had M1c disease. The median OS for patients was M0, 12 years (95% CI: 9.4–NA); M1a, 2.2 years (95% CI: 1.0–NA); M1b, 2.0 years (95% CI: 1.5–2.7); and M1c, 0.9 years (95% CI: 0.78–0.99). The HRs for patients were M1a, 2.69 (95% CI: 1.77–4.08); M1b, 2.83 (95% CI: 2.35–3.42); and M1c, 4.38 (95% CI: 3.80–5.05). In terms of HRs, patients with M1a, M1b, and M1c disease have significantly higher HRs compared with patients with M0 disease (*p* < 0.001 for all comparisons) (Figure 1).

### 3.3. Survival Outcomes of Advanced Melanoma Classified According to Alternative Criteria

**Figure 2:**
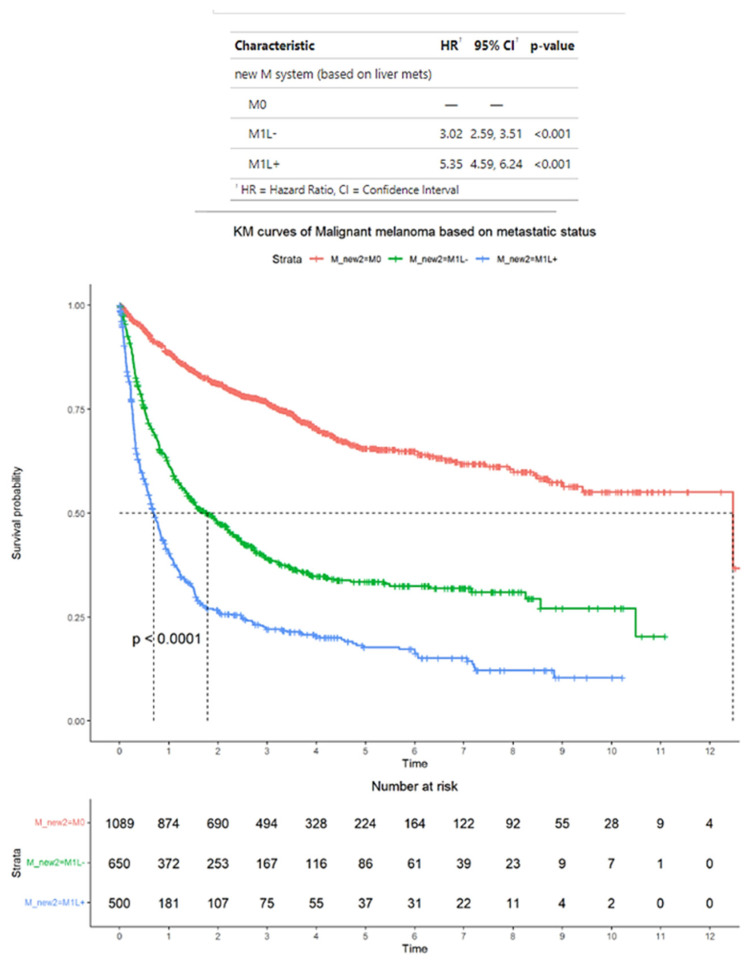
Survival outcomes in the entire sample of patients with advanced melanoma classified into those without distant metastases (M0), those with distant metastases with liver metastases (M1L+), and those with distant metastases without liver metastases (M1L−). Time is measured in years.

As an alternative to the AJCC staging criteria, patients with M1 disease were classified based on whether they have M1 disease spread to the liver (M1L+) or not (M1L−). According to these alternative criteria, of the total 2239 patients with advanced melanoma, 1089 patients had M0 disease, 650 patients had M1L− disease, and 500 patients had M1L+ disease. The median OS for patients was M0, 12 years (95% CI: 9.4–NA); M1L−, 1.8 years (95% CI: 1.4–2.2); and M1L+, 0.69 years (95% CI: 0.60–0.82). The HRs for patients wereM1L−, 3.02 (95% CI: 2.59–3.51) and M1L+, 5.35 (95% CI: 4.59–6.24). In terms of HRs, patients with M1L− and M1L+ disease have significantly higher HRs compared with patients with M0 disease (*p* < 0.001 for all comparisons) (Figure 2).

### 3.4. Survival Outcomes in the Subset of Advanced Melanoma Treated with Immunotherapy Classified According to AJCC Staging Criteria

**Figure 3:**
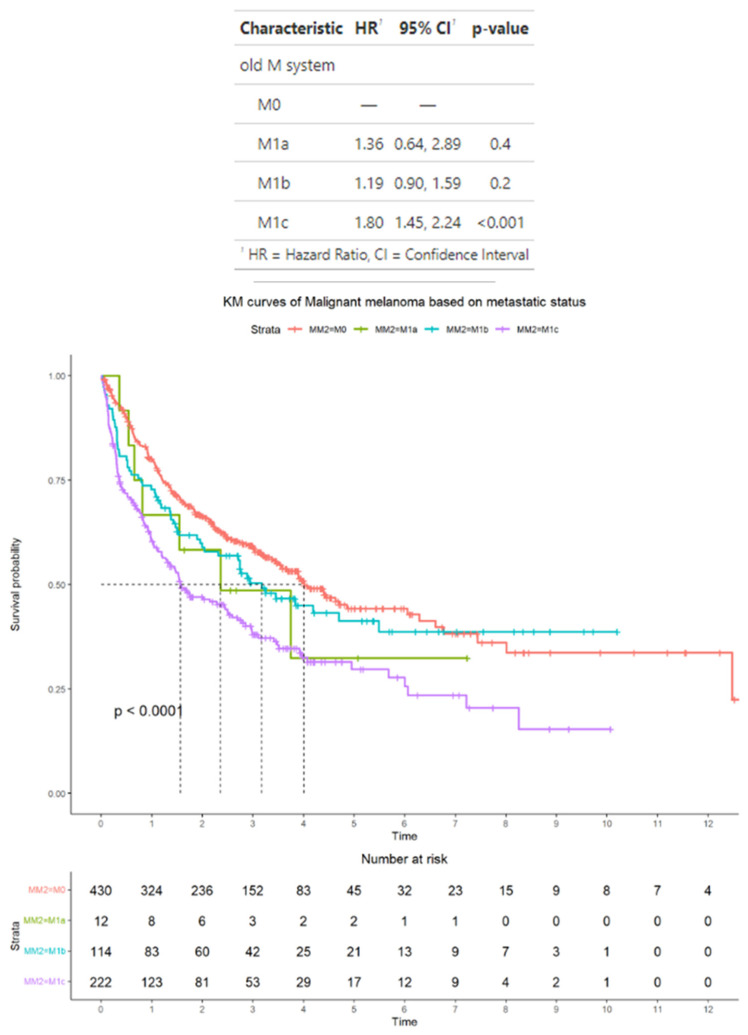
Survival outcomes in the subset of patients with advanced melanoma treated by immunotherapy and classified according to American Joint Committee on Cancer staging criteria. Time is measured in years.

In the subset of 778 patients who were treated with immunotherapy, according to AJCC staging criteria, 430 patients had M0 disease, 12 patients had M1a disease, 114 patients had M1b disease, and 222 patients had M1c disease. In this subset, the median OS for patients was M0, 4 years (95% CI: 3.5–6.3); M1a, 2.4 years (95% CI: 0.6–2.9); M1b, 3.1 years (95% CI: 0.9–1.6); and M1c, 1.6 years (95% CI: 1.4–2.2). M1a, 1.36 (95% CI: 0.64–2.89); M1b, 1.19 (95% CI: 0.90–1.59); and M1c, 1.80 (95% CI: 1.45–2.24). In terms of HRs, only patients with M1c disease have a significantly higher median HR compared with patients with M0 disease (*p* < 0.001) (Figure 3).

### 3.5. Survival Outcomes in the Subset of Advanced Melanoma Treated with Immunotherapy Classified According to Alternative Criteria

**Figure 4:**
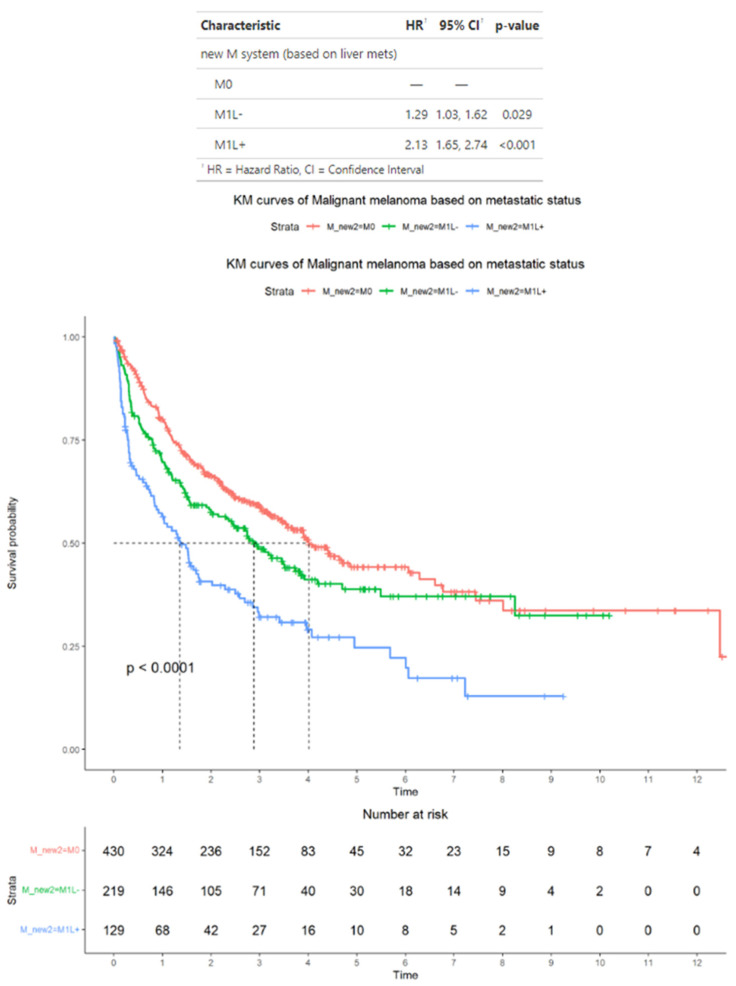
Survival outcomes in the subset of patients with advanced melanoma treated by immunotherapy and classified into those without distant metastases (M0), those with distant metastases with liver metastases (M1L+), and those with distant metastases without liver metastases (M1L−). Time is measured in years.

In the subset of 778 patients who were treated with immunotherapy, as an alternative to the AJCC staging criteria, patients with M1 disease were classified based on whether they had M1 disease spread to the liver (M1L+) or not (M1L−). According to these alternative criteria, 430 patients had M0 disease, 203 patients had M1L− disease, and 145 patients had M1L+ disease. In this subset, the median OS for patients was M0, 4 years (95% CI: 3.5–6.3); M1L−, 2.9 years (95% CI: 2.3–3.9); and M1L+, 1.4 years (95% CI: 0.92–2.0). The HRs for patients were M1L−, 1.29 (95% CI: 1.03−1.62) and M1L+, 2.13 (95% CI: 1.65−2.64). In terms of HRs, only patients with M1L+ disease have significantly higher median HR compared with patients with M0 disease (*p* < 0.001) (Figure 4).

### 3.6. Survival Outcomes in the Subset of Advanced Melanoma Treated with Immunotherapy and with BRAF Mutation Status Available

**Figure 5:**
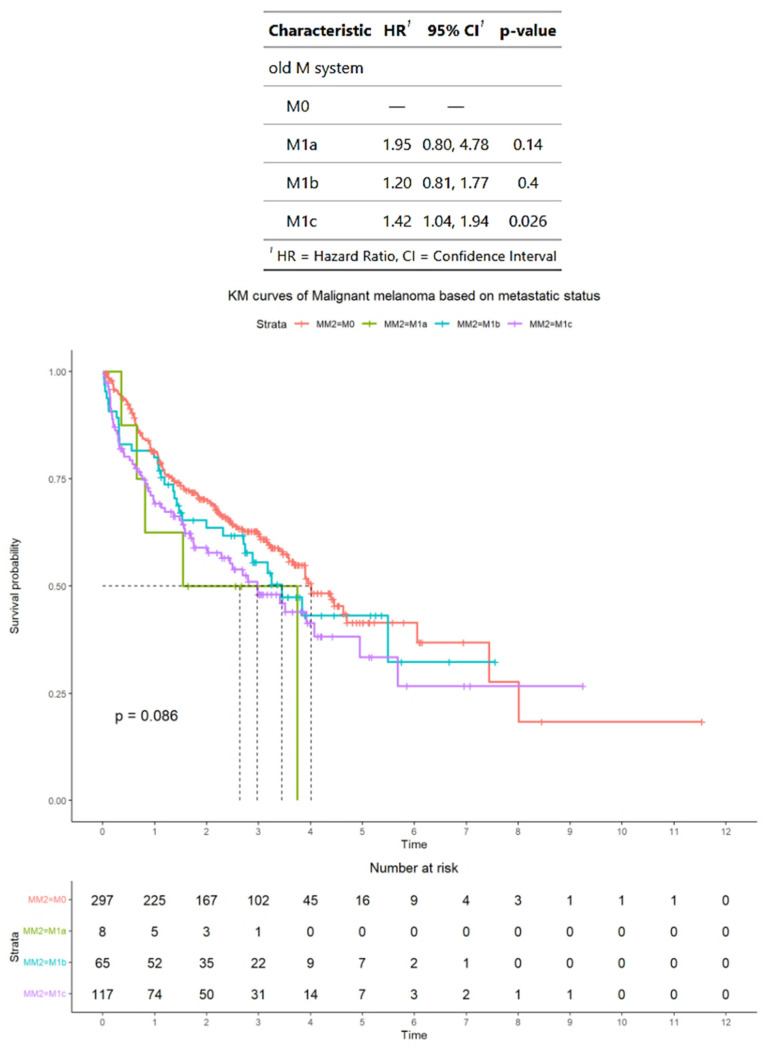
Survival outcomes in the subset of patients with advanced melanoma treated by immunotherapy and who have *BRAF* mutation status available, classified according to American Joint Committee on Cancer staging criteria. Time is measured in years.

In the subset of 487 patients who were treated with immunotherapy and who had *BRAF* mutation status available, according to AJCC staging criteria, 297 patients had M0 disease, 8 patients had M1a disease, 65 patients had M1b disease, and 117 patients had M1c disease. The median OS for patients was M0, 4 years (95% CI: 3.6–NA); M1a, 2.6 years (95% CI: 0.8–NA); M1b, 3.4 years (95% CI: 2.7–NA); and M1c, 3.0 years (95% CI: 2.0–5.7). The HRs for patients were M1a, 1.95 (95% CI: 0.80–4.78); M1b, 1.20 (95% CI: 0.81–1.77); and M1c, 1.42 (95% CI: 1.04–1.94). In terms of HRs, only patients with M1c disease have a significantly higher median HR compared with patients with M0 disease (*p* < 0.03) (Figure 5).

**Figure 6:**
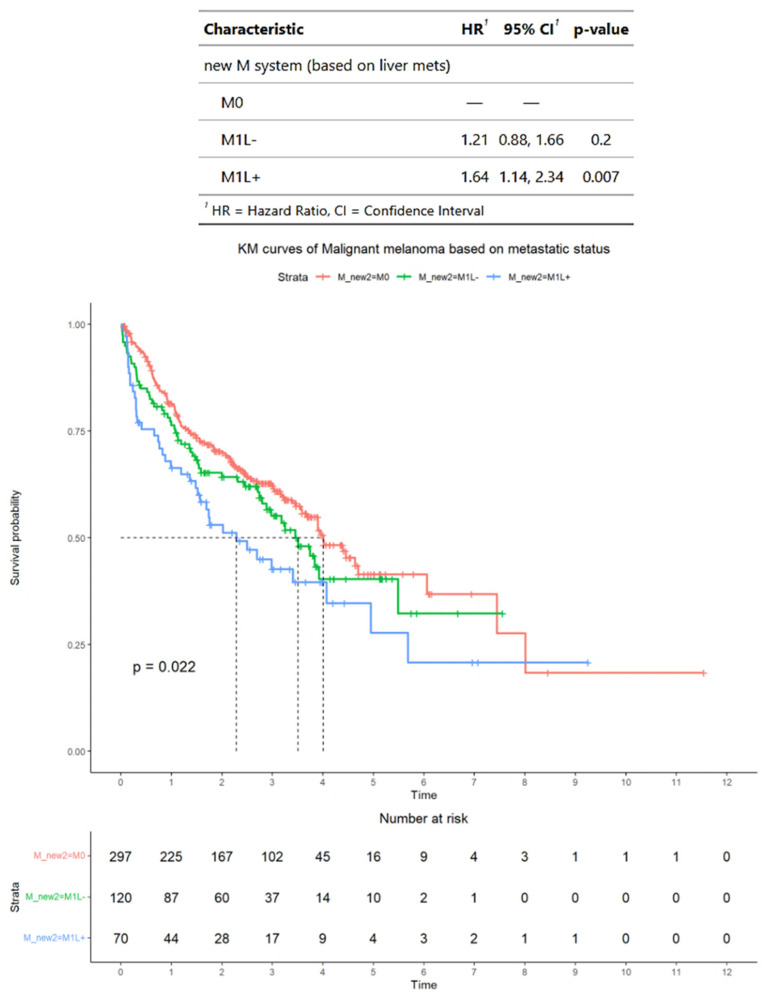
Survival outcomes in the subset of patients with advanced melanoma treated by immunotherapy and who have *BRAF* mutation status available, classified into those without distant metastases (M0), those with distant metastases with liver metastases (M1L+), and those with distant metastases with liver metastases (M1L−). Time is measured in years.

Meanwhile, in this subset, according to alternative criteria, 297 patients had M0 disease, 120 patients had M1L− disease, and 70 patients had M1L+ disease. The median OS for patients was M1L−, 3.5 years (95% CI: 2.8-NA) and M1L+, 2.3 years (95% CI: 1.5–5.7). The median HRs for patients were M1L−, 1.21 (95% CI: 0.88–1.66) and M1L+, 1.64 (95% CI: 1.14–2.34). In terms of HRs, only patients with M1L+ disease have significantly higher median HR compared with patients with M0 disease (*p* = 0.007) (Figure 6).

### 3.7. Survival Outcomes Comparing M1b, M1bL−, and M1cL+ Disease

**Figure 7:**
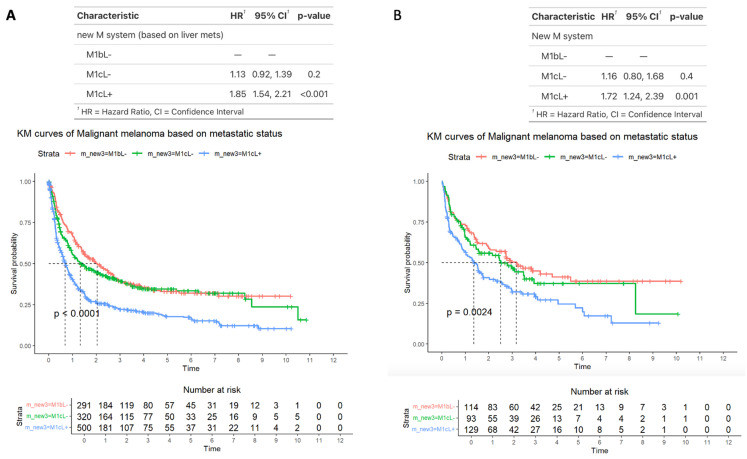
Survival outcomes in a subset of patients with advanced melanoma comparing patients with M1b disease (by definition those without liver metastases [M1bL−]), M1C disease without liver metastases (M1cL−), and M1c disease with liver metastases (M1cL+), across all patients (**A**) or across patients who underwent immunotherapy (**B**). Time is measured in years.

Lastly, we performed a subset analysis comparing patients with M1b disease (by definition those without liver metastases [M1bL−]), M1C disease without liver metastases (M1cL−), and M1c disease with liver metastases (M1cL+). The median OS for patients was for M1bL−, 2 years (95% CI: 1.5–2.7); for M1cL−, 1.3 years (95% CI: 1.0–2.1); and for M1cL+, 0.69 years (95% CI: 0.6–0.82). The HRs for patients were M1cL−, 1.13 (95% CI: 0.92–1.39) and M1cL+, 1.85 (95% CI: 1.54–2.21). In terms of HRs, only patients with M1cL+ disease have significantly higher median HR compared with patients with M1bL− disease (*p* < 0.001) (Figure 7A). This was also the case for the 336 patients who received immunotherapy (*p* = 0.001) (Figure 7B). For those on immunotherapy with BRAF status available (n = 177), 76 patients had altered BRAF, and 101 patients had wild type BRAF. No significant differences in survival were found for either subpopulation when stratified by M1cL- and M1cL+ (for altered BRAF, M1cL+ HR 1.40 (95% CI: 0.6–3.1, *p* = 0.4), and for wild-type BRAF, M1cL+ HR 1.43 (95% CI: 0.8–2.6, *p* = 0.2).

## 4. Discussion

This study used NLP to perform large-scale labeling of data from the CT radiology reports of 2239 patients with advanced melanoma to assess the relationship between the site of metastases and survival, and particularly, if liver metastases portend poorer prognosis. The results showed that between M0, M1a, M1b, and M1c disease (i.e., classifications according to the AJCC staging criteria), survival outcomes were poorest for patients with M1c disease (distant metastases to non-CNS visceral sites), in whom the median OS was 0.9 years and the median HR was 4.38 (*p* < 0.001 compared with M0 disease). Furthermore, between M0, M1L+, and M1L− disease (i.e., classifications according to an alternative criterion), survival outcomes were poorest for patients with M1L+ disease (distant metastases to the liver particularly), in whom the median OS was 1.2 years and the median HR was 5.35 (*p* < 0.001) compared with M0 disease. Similarly to the entire patient population, in the subset of patients treated with immunotherapy, those with M1c and M1L+ disease had the poorest survival outcomes, with a median OS of 1.6 years and 1.2 years, respectively, and a median HR of 1.80 and 2.13 (*p* < 0.01 for both compared with M0 disease), albeit these patients had better survival outcomes compared with patients in the entire population, including those not treated with immunotherapy. Overall, whether AJCC or alternative criteria were used, patients with M1L+ disease had the poorest outcomes, as shown by their shortest median OS and highest median HR compared with other patients.

The life expectancy of patients with advanced melanoma has been improved by the advent of checkpoint inhibitors over the past decade. However, our results suggest that patients with liver metastases may derive reduced benefit from immunotherapy. The liver contains a high density of tolerogenic cytokines as well as T-regulatory cells and plays an immunoregulatory role, which can be affected by the presence of hepatic metastases [25]. Indeed, in preclinical models, it was noted that the microenvironment of liver metastases resulted in immunosuppression associated with a reduction in the number of activated CD8^+^ T cells [24]. Further, in a study involving patients with melanoma and lung cancer, it was noted that patients with liver metastases had diminished tumoral T-cell diversity and function and lower CD8^+^ T-cell density at the invasive tumor margin than patients without liver metastases [26]. When we performed a further subset analysis comparing patients with M1cL+ disease and M1cL− disease, patients with M1cL+ disease had poorer survival outcomes compared with patients with M1cL− disease (M1cL+: median OS, 1.3 years, median HR, 1.85; M1cL−: median OS, 0.8, median HR, 1.13), further supporting the hypothesis that liver metastases in particular are prognostic of poorer survival.

Our results are consistent with several studies in the literature. Pires da Silva et al. [27] demonstrated that in patients with melanoma who received immunotherapy, the different sites of metastatic disease impacted survival, with patients with liver metastases experiencing an inferior clinical response. Yu et al. [11] examined an institutional sample of 718 patients, including a subset of 182 patients with metastatic melanoma who received ICB immunotherapy, and noted that patients with liver metastases had significantly less clinical benefit from immunotherapy. Rubatto et al. [28] performed a retrospective analysis of 190 patients with advanced metastatic melanoma who received ICB immunotherapy as monotherapy, including almost one-third who had liver metastases at the initiation of treatment. Of those with liver metastases, the response rate was lower (27%) than in those patients without liver metastases (73%). In addition, the median OS was lower in patients with liver metastases (8 months) compared with those without liver metastases (29 months). Najjar et al. [29] conducted a multicenter retrospective analysis of 89 patients with uveal melanoma, of whom 83/89 (93%) had liver metastases, to assess the efficacy and safety of dual checkpoint blockade with nivolumab and ipilimumab. They found a median OS of 15 months, which was similar to the OS found in our study of 1.2 years. Similarly, Piulats et al. [30] aimed to assess the efficacy of combination ICB therapy as first-line therapy in 52 patients with metastatic uveal melanoma who were not eligible for liver resection and found that the median OS was 12.7 months. Bilen et al. [12] conducted a retrospective review of 90 patients treated with immunotherapy in phase 1 clinical trials, including one-third of patients (33%) with melanoma. The median OS was longer for patients without liver metastases than for those with liver metastases (21.9 vs. 8.1 months; *p* = 0.0048), consistent with our findings. In addition, Bethof et al. [13] retrospectively evaluated 254 patients with metastatic melanoma treated with immunotherapy to determine factors associated with OS and found that the presence of liver metastasis at the time of first presentation to an oncologist doubled the hazard for death (HR: 2.1 years (95% CI: 1.3–3.2); *p*  = 0.001), also in line with our analysis. More recently, Dercle et al. [31] applied machine learning algorithms to baseline medical data to estimate the impact of immunotherapy on OS in 695 patients with advanced cancer, most with melanoma (n = 102, 41%). They found that the presence of liver metastases (LM+) at baseline was an independent predictor of OS when present with elevated LDH compared with patients without liver metastases (LM−) with normal/low LDH (median OS: 3.1 vs. 15.3 months; *p* < 0.0001). They concluded that LM+ status could be used to identify patients who may not benefit from immunotherapy.

Notably, in the subset of patients treated with immunotherapy, the difference in OS between patients with liver metastases with and without *BRAF* mutation status (3.5 vs 2.2 years, respectively) suggests that the combined use of ICB drugs and *BRAF*/*MEK* inhibitors may improve survival outcomes, in line with previous studies [8,24].

This study has some limitations. Firstly, our sample was evaluated retrospectively and is relatively small, especially with regard to the subset of patients whose *BRAF* mutation status was available. This is due in part to the relatively recent FDA approval of MSK-IMPACT testing, which occurred in November 2017, leading to a bias towards more recently treated patients. Secondly, we do not have data to determine if patients with M0 disease also included patients with unresectable M1 stage III disease, typically treated with systemic therapy. Thirdly, patients were not classified into those with M1d (distant metastatic disease to CNS with or without M1a, M1b, or M1c sites of disease), as the NLP model was limited to CT reports of the body only, and so the predictive value of brain metastases could not be adequately analyzed. Fourth, our previously validated NLP models [14] do not have 100% accuracy. However, the purpose of using NLP is not to achieve 100% accuracy, as manual curation is also prone to human error, but to rapidly extract structured data at scale to perform this type of analysis. Fifth, we did not perform subset analysis of patients undergoing different immunotherapy regimens, an important area of further research to see which subset of patients with liver metastases may have the most clinical benefit. LDH values, which are strongly associated with prognosis in patients with advanced melanoma, were not available for most patients at the time of baseline CT imaging and so were not included in our data analysis. The number of patients with available BRAF mutation status was also limited, and further studies incorporating these and other clinical variables are warranted.

## 5. Conclusions

In conclusion, patients with liver metastases demonstrated inferior survival when treated with immunotherapy when compared with other patients with other visceral sites of metastatic disease. These results provide additional support for clinicians to provide combinatorial treatments such as hepatic-directed therapy in combination with immunotherapy when liver metastases are present. NLP is a useful tool to rapidly investigate prognosis of melanoma patients on immunotherapy with different metastatic patterns, and additional study in this area is warranted.

## Data Availability

Data generated or analyzed during the study are available from the corresponding author by request.

## Abbreviations

The following abbreviations are used in this manuscript:

CNS	central nervous system
CT CAP	CT of the chest, abdomen, and pelvis
HR	hazard ratio
ICB	immune checkpoint blockade
OS	overall survival
NLP	natural language processing
